# Prevalence of *Fusarium* Wilt Disease of Cucumber (*Cucumis sativus* Linn) in Peninsular Malaysia Caused by *Fusarium oxysporum* and *F. solani*

**DOI:** 10.21315/tlsr2020.31.3.3

**Published:** 2020-10-15

**Authors:** Hazirah Mohd Din, Osamah Rashed, Khairulmazmi Ahmad

**Affiliations:** 1 Department of Plant Protection, Faculty of Agriculture, Universiti Putra Malaysia, 43400 Serdang, Selangor, Malaysia; 2 Institute of Plantation Studies, Universiti Putra Malaysia, 43400 Serdang, Selangor, Malaysia

**Keywords:** Disease Prevalence, Cucumber, *Fusarium oxysporum*, *Fusarium solani*, Sebaran Penyakit, Timun, *Fusarium oxysporum*, *Fusarium solani*

## Abstract

*Fusarium* wilt disease is one of the most problematic and destructive disease in cucumber production. The causative agents are *Fusarium oxysporum* and *F. solani*. These pathogens are soil borne and transmitted through infested soil and water. A field survey was conducted to study the disease prevalence in the major growing areas of cucumber in Peninsular Malaysia. Field study revealed that the disease was highly prevalence in the field with the disease incidence was in the range of 10%–60%. The morphological properties of *F*. *oxysporum* are microconidia (3.8–15.7 μm × 2.9–4.9 μm), macroconidia (14.8–38.5 μm × 2.4–5.7 μm) and number of septate was 1–4. While for *F. solani* are microconidia (3.39–14.63 μm × 2.36–4.44 μm), macroconidia (7.22–50.46 μm × 2.43–6.14 μm) and number of septate was 1–5. Based on molecular identification had confirmed that the disease is caused by *F. oxysporum* and *F. solani* with similarity index of 99%–100% based on internal transcribed spacer (ITS) gene sequences. The pathogenicity test showed that the symptoms of *Fusarium* wilt disease was firstly appeared as yellowing of old leaves. Progressively, the infected plant will be wilted and finally died. The outputs of this study are highly important to establish an effective disease management programme to reduce disease prevalence and yield loss in the field.

## Abstract (ms)

Penyakit layu *Fusarium* adalah salah satu daripada penyakit timun yang sangat bermasalah dan merosakkan. Agen penyebab kepada masalah ini ialah *Fusarium oxysporum* dan *F. solani*. Patogen ini ialah bawaan tanah dan boleh disebarkan melalui tanah dan air yang tercemar. Pemantauan ladang telah dilaksanakan untuk mengkaji kejadian penyakit ini di beberapa kawasan penanaman utama di Semenanjung Malaysia. Dapatan kajian lapangan mendedahkan bahawa kejadian penyakit amat tinggi di ladang dengan peratus kejadian penyakit di antara 10%–60%. Ciri-ciri morfologi *F. oxysporum* ialah mikrokonidia (3.8–15.7 μm × 2.9–4.9 μm), makrokonidia (14.8–38.5 μm × 2.4–5.7 μm), bilangan septa adalah 1–4. Manakala bagi *F. solani* mikrokonidia (3.39–14.63 μm × 2.36–4.44 μm), makrokonidia (7.22–50.46 μm × 2.43–6.14 μm) dan bilangan septa adalah 1–5. Berdasarkan pengenalpastian molekular telah disahkan bahawa penyakit tersebut disebabkan oleh *F. oxysporum* dan *F. solani*, dengan indek persamaan di antara 99%–100% berdasarkan jujukan gen ITS. Ujian patogenisiti pula menunjukkan bahawa simptom awal penyakit layu *Fusarium* adalah kekuningan pada daun tua. Selanjutnya pokok terjangkit akan menjadi layu dan akhirnya akan mati. Hasil dapatan kajian ini sangat penting untuk membangunkan program pengurusan penyakit berkesan demi mengurangkan kejadian penyakit dan kehilangan hasil ladang.

**Highlights**

- *Fusarium* wilt disease is one of the most destructive diseases in cucumber.
- *Fusarium solani* and *Fusarium oxysporum* are the causative agents of the disease.
- *Fusarium* wilt disease of cucumber is highly prevalent in the field (10%–60% of disease incidence) with limited disease control.

## INTRODUCTION

Cucumber (*Cucumis sativus* Linn) is a vegetable crop with annual production of USD9.76 billion worldwide. Cucumber is largely planted and produced in Asia (87.2%) with China is dominated the word cucumber production (Meng *et al*. 2018). Cucumber is also an important fruit crop species cultivated both in greenhouse and field in Malaysia, major production areas are Johor, Perak and Terengganu. Owing to the limited availability of agricultural land and high market demand, continuous cucumber cropping has been widely adopted by majority farmers. Continuous cropping affects cucumber yield and quality. Soil-borne disease pathogens and secondary salinisation are commonly observed in continuously cropped soil (Meng *et al*. 2018).

Nonetheless, one of the major constraints in cucumber cultivation and dropping of yield are mostly due to phytopathogens. The pathogens caused deterioration of yield by reducing the quality and quantity of the crop produces. Various cucumber diseases were reported such as downy mildew (*Pseudoperonospora cubensis*), powdery mildew (*Erysiphe cichoracearum*), gray mold (*Botrytis cinerea*), root rot (*Phomopsis sclerotioides*), white mold (*Sclerotinia sclerotiorum*), gummy stem blight and black rot (*Didymella bryoniae*), anthracnose (*Colletotrichum orbiculare*) and *Fusarium* wilt (*Fusarium oxysporum*). However, the most problematic and destructive disease in cucumber production is *Fusarium* wilt. This disease is caused by *Fusarium oxysporum* f. sp. *cucumerinum* (Ye *et al*. 2004). It is a soil borne disease and transmitted through soil and water. This disease is a major soil dweller that leads to severe shortage in cucumber supply under greenhouse condition (Shen *et al*. 2008). The fungus can attack susceptible plants at any stage of growth. Infection of the hypocotyls of young plants can result in pre- or post-emergence damping-off. Infection in older plants can cause yellowing starts from old leaves, stunting or wilting. Once wilting occurs, death may result in three to five days. Affected plants may develop a lesion on the lower stem. The fungus affects the vascular system and the infected plants may not show any noticeable symptoms until they begin bearing fruits. Browning of vascular system is usually evident in lower stem, crown and tap root. After the plant dies, a white mycelium mat forms on external plant surfaces (Pscheidt & Ocamb 2016). As a result, the fungus can infect cucumber plant at any stages causing high loss of yield in cucumber productions.

For the Malaysia case, *Fusarium* wilt of cucumber has not been extensively studied. To the best of our knowledge, there was only one research publication reported. However, the study was very general and brief. So that, comprehensive research, accurate and fast identification method of the pathogen is highly necessary for appropriate management of the disease. Comprehensive study is urgently needed because the disease is widely distributed with high disease occurrences. The present study was conducted with the aims of to evaluate on the disease prevalence of *Fusarium* wilt of cucumber in Peninsular Malaysia and to isolate and identify the causative agents of *Fusarium* wilt disease for effective disease management in the field.

## MATERIALS AND METHODS

### Field Disease Assessment and Sample Collection

Field survey for *Fusarium* wilt disease of cucumber was conducted at the major growing areas in Peninsular Malaysia namely Selangor (Sepang), Perak (Sungai Siput and Batu Gajah), Pahang (Cameron Highland), Johor (Kluang) in 2016. Field disease assessments such as field disease symptoms and disease incidence (%) was conducted by random sampling method of at least 100 plants were evaluated per farm. Farm disease incidence was recorded as percentage of disease incidence based on the total number of infected plants over total number of surveyed plants. About 30 infected roots were collected per farm and the specimens were brought to Microbiology Laboratory, Department of Plant Protection, Universiti Putra Malaysia for pathogen isolation and identification.

### Isolation and Morphological Characterisation of *Fusarium* sp

*Fusarium* spp. were isolated using PCNB (Pentachloronitrobenzene) media and characterisation as was described by Booth (1971) and Leslie and Summerell (2006). Pure culture was obtained from a single spore technique and were grown on PDA and Spezieller Nährstoffarmer Agar (SNA). Potatoes Dextrose Agar (PDA) media was used to assess colony colour, texture and growth rate. Meanwhile, SNA media was used to examine the formation and types of macroconidia, microconidia and conidiogeneous cells. Number of septa, conidial length and width of 30 spores per each isolate and were measured using eye piece micrometer and compound light microscope. For cultural properties, three replicates were prepared for each isolate. The growth of each isolate was measured at 3rd, 5th and 7th day after incubation. The data was recorded and analysed using SAS software 9.4 to determine any variations among isolates.

### Pathogenicity Test

Pathogenicity test was conducted at a glasshouse in Ladang 16, Faculty of Agriculture, Universiti Putra Malaysia. In total, 12 isolates of both *Fusarium oxysporum* and *F. solani* were tested. Local cucumber variety (cucumber 107) supplied by MARDI was used. Ten seedlings per treatment with the age of 2 week-old were inoculated using spore suspension at concentration of 1 × 10^6^ spores mL^−1^ and was planted in a polybag (10 cm width × 12 cm depth) using sterilised soil mixture with ratio of 3: 2: 1 (soil: sand: organic compound) as a media. The inoculation was conducted by injuring the lower part of the stem and drenched with 50 mL of spore suspension of respected isolates onto tested seedlings. For negative control treatment, 50 mL of sterile distilled water was used. To assess the aggressiveness of the isolates, data of the area under disease progress curve (AUDPC) and disease severity index (DSI) (Madden *et al*. 2007) and disease symptoms were recorded from the first week of inoculation until fourth week of inoculation period. The experimental design used was completely randomise design (CRD) with 13 treatments and 10 replications. The data was carefully recorded and analysed using MATLAB software to determine any mean variations among treatments.

### Molecular Identification of *Fusarium* spp

Internal transcribed spacer (ITS) gene of *Fusarium* sp. was extracted using DNeasy Plant Mini kits protocol. The universal primer pairs of ITS1 and ITS4 were used for PCR amplification and gene sequencing. The nucleotide sequences were: ITS1 (5’-TCCGTAGGTGAACCTGCGG-3’) and ITS4 (5’-TCCTCCGCTTATTGATATGC-3’) (White *et al*. 1990). Amplification reactions were prepared to a total volume of 30 μL containing 2 μL DNA templates, 1.5 μL forward primers, 1.5 μL reverse primers, 15 μL PCR mastermix (Taq DNA polymerase, BIOMAX Company) and 10 μL of nuclease free water. After mixing the reagents, the amplification reactions were vortexed lightly for 30 s. The PCR amplifications were performed in VITAR SEGATEC-Bio Rad Company Thermocycler. Thermal cycling consisted of initial denaturation for 4 min at 95°C, followed by 40 cycles denaturation at 95°C for 30 s, annealing at 58.8°C for 30 s and extension 72°C for 1 min and final extension of 72°C for 5 min and keeping time at 4°C. Agarose gel (1%) was prepared by adding 1 g agarose with 100 mL of 1x TBE buffer (PH8.0) and 4 μL of red-safe stain was added. The tray was placed into the tank containing 1x TBE buffer. About 10 μL of ladder (100bp DNA Ladder H3 RTU, 1st base company, Malaysia) was used as molecular marker to determine the size of the amplified DNA bands. Ladder was loaded in the first well followed by loading 7 μL of PCR product samples. Electrophoresis was carried out at 80 V cm^−1^ for 60 min. Gel was visualised under UV light using gel documentation system (6.03, Syngene Laboratories).

### Gene Sequencing Analysis

PCR products were sent to MyTACG Bioscience Enterprise for purification and sequencing of DNA. The purified products of PCR were sequenced using automated DNA sequencer (ABI PRISM^®^). The ITS gene sequences were aligned using Bioedit software version 7.2 (http://www.mbio.ncsu.edu/bioEdit/bioEdit). All sequences were compared to sequences of *Fusarium* species available in the Genbank database using BLAST network services similarities present in NCBI database (National Centre for Biotechnology Information) (http://www.ncbi.nlm.nih.gov).

## RESULTS

### Field Disease Prevalence

**Figure 1:**
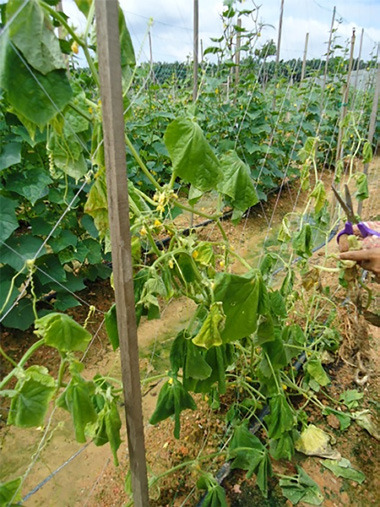

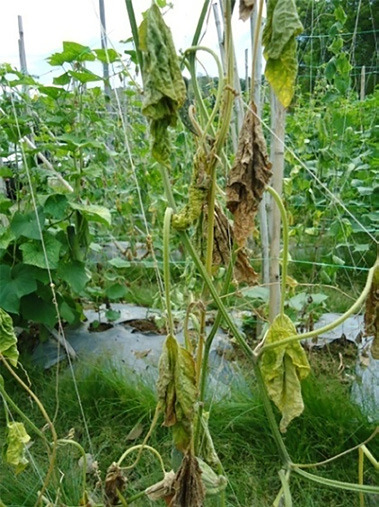

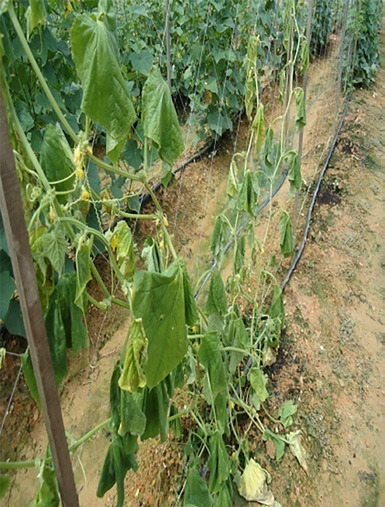

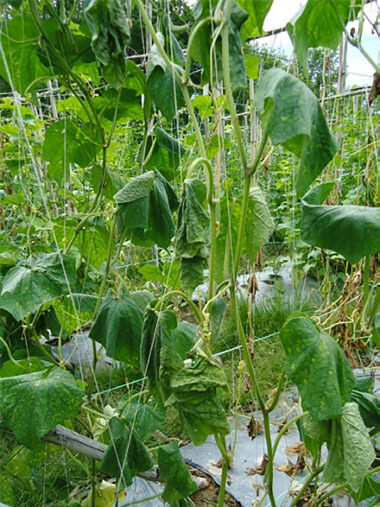

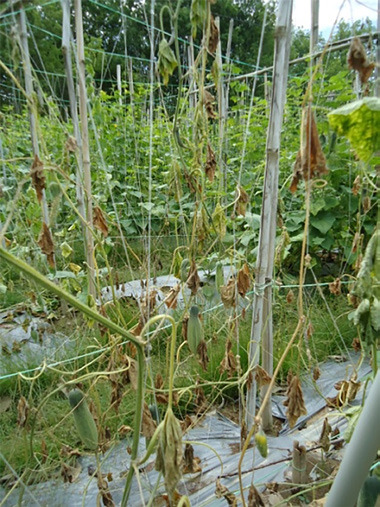
Field symptoms of *Fusarium* wilt disease observed in sampling areas in Perak. (A) Cucumber plants showing yellowing leaves and wilting; (B, C and D) Cucumber plants showing *Fusarium* wilt disease symptoms of wilting plant; (E) Cucumber plants showing plant dead symptom.

*Fusarium* wilt disease symptoms were observed in infected surveyed areas during the respective period. From the survey data, typical *Fusarium* wilt symptoms i.e. leaf yellowing and severe wilting were recorded at all surveyed areas (Fig. 1). Field data of disease incidence and disease symptoms were summarised in Table 1. Findings revealed that field disease incidence was in the range of 10%–60%. The areas with the highest disease incident was Sungai Siput, Perak (60%) while Kluang, Johor was the lowest disease incidence (10%). This data indicated that the disease was prevalent particularly on mature tree. However, the disease was also detected on young tree with low disease incidence.

### Morphological and Cultural Characteristics of *Fusarium* spp

**Figure 2:**
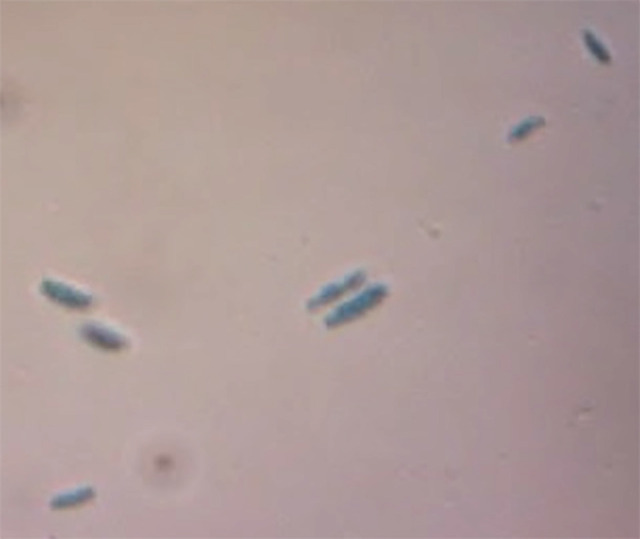

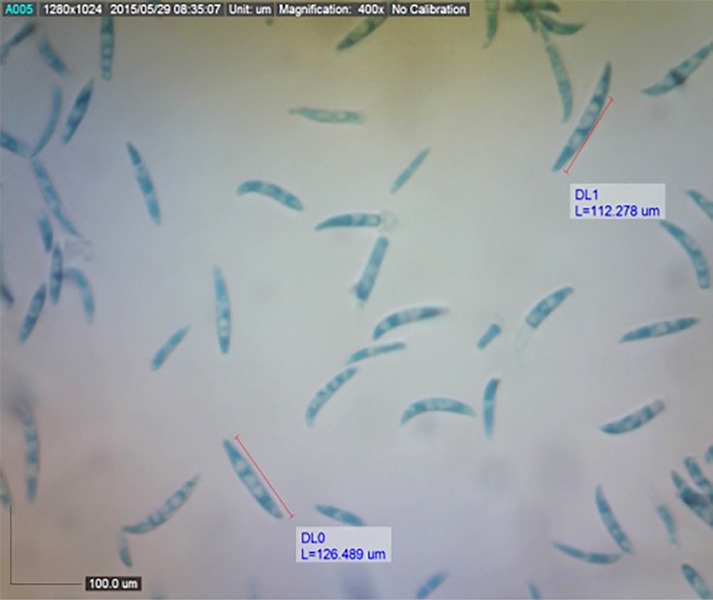

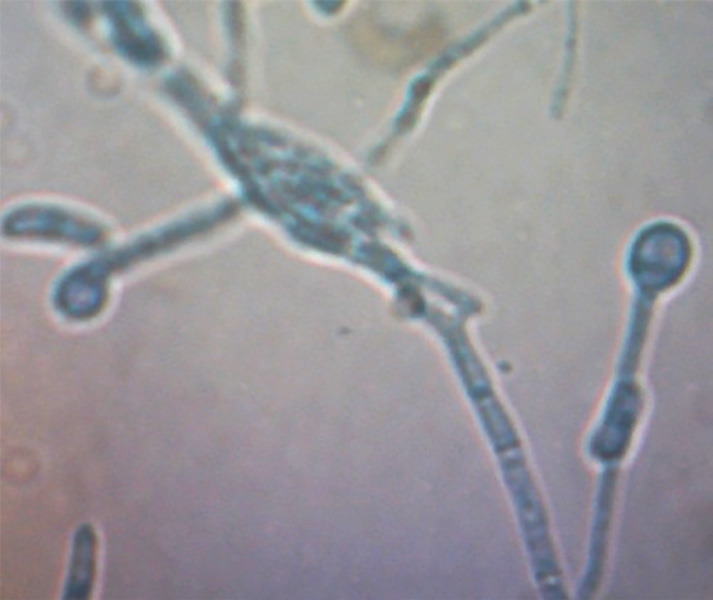

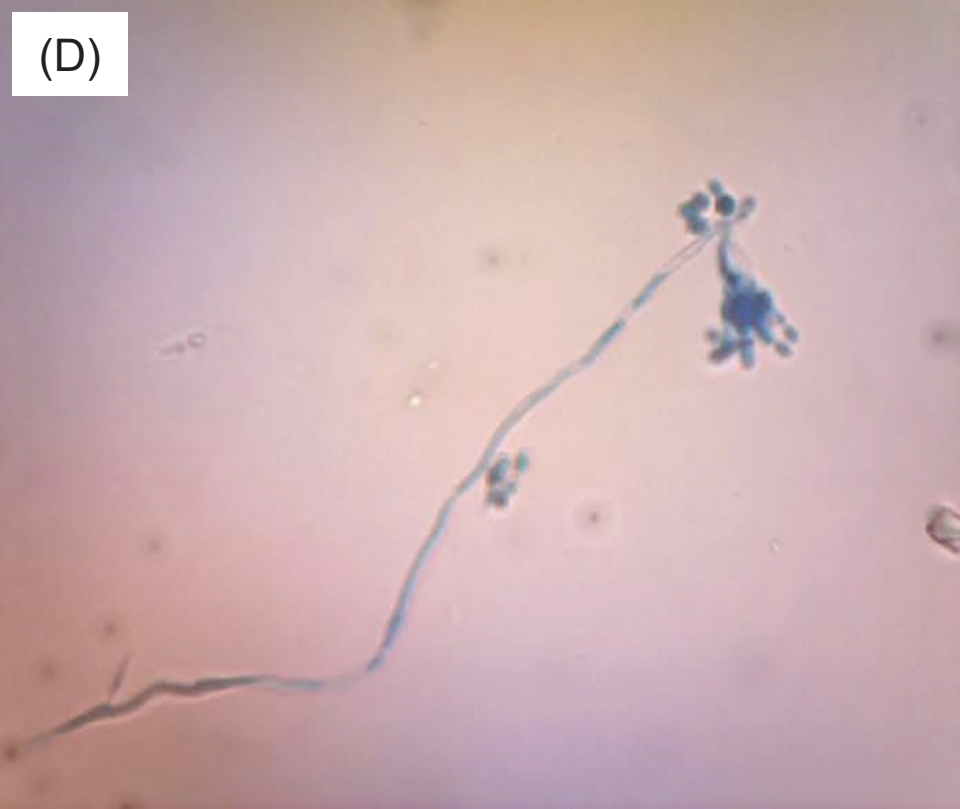
Morphological features of *Fusarium oxysporum* grown on SNA media. (A) Microconidia; (B) Macroconidia; (C) Chlamydospore; (D) False heads on short monophialides.

**Figure 3:**
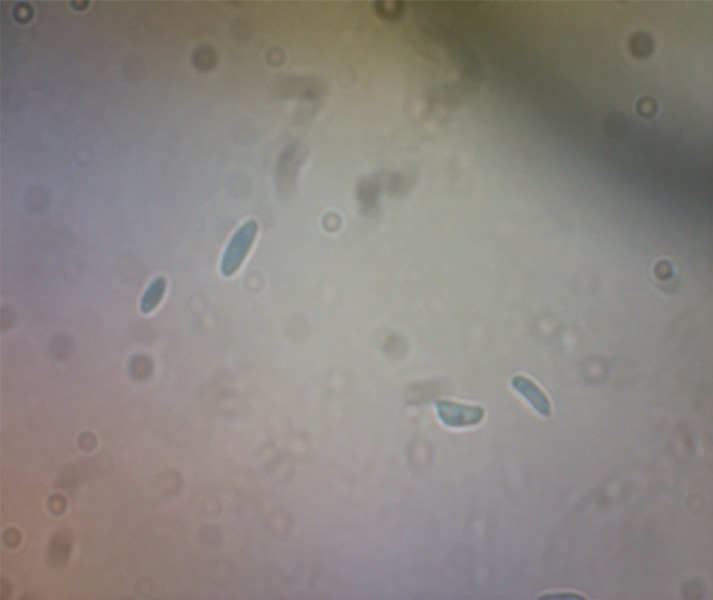

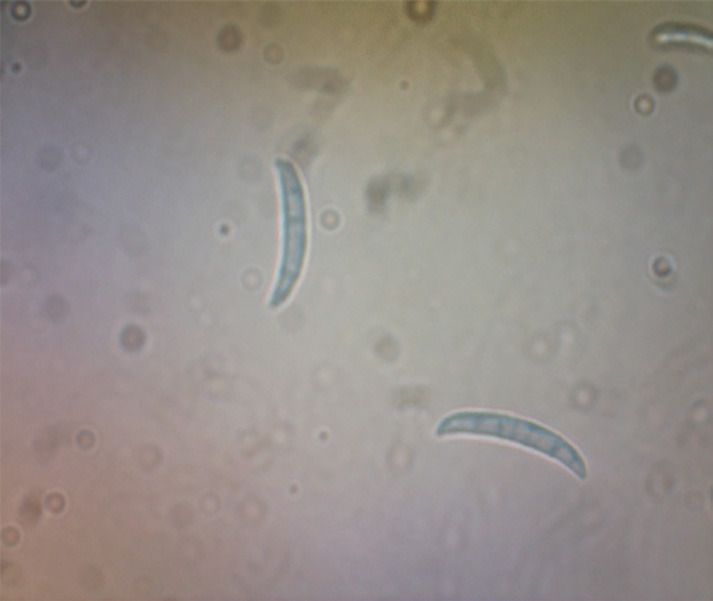

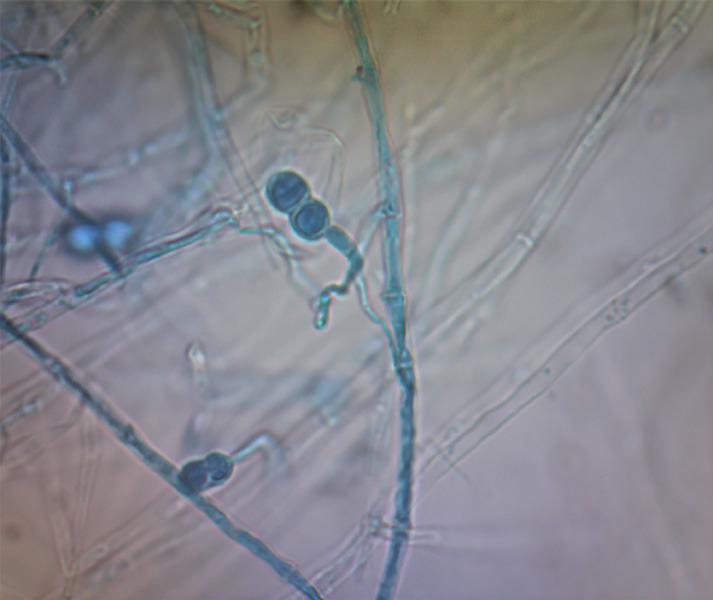

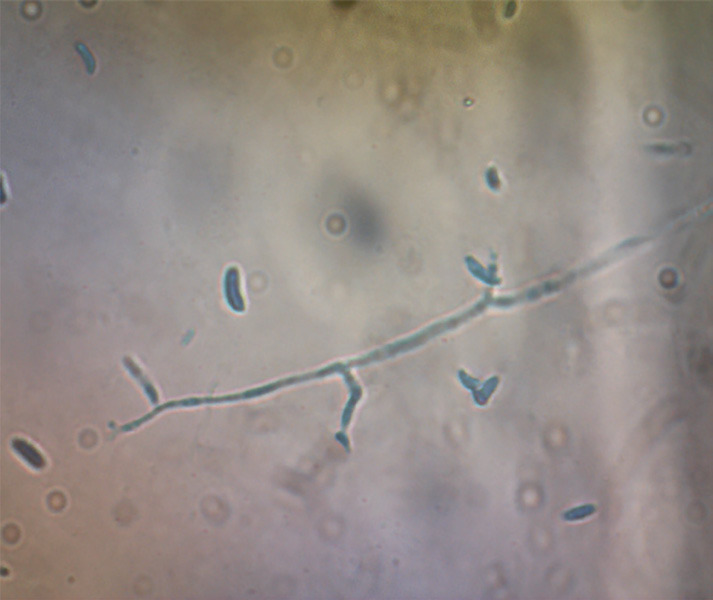
Morphological features of *Fusarium solani* grown on SNA media. (A) Microconidia; (B) Macroconidia; (C) Chlamydospore; (D) Short monophialides.

Based on morphological characteristics, two species of *Fusarium* were identified. Five isolates were *F. oxysporum* while the rests of the isolates were *F. solani*. Tables 2–4 and Fig. 2 demonstrated the morphological characteristics of *F. oxysporum*. Meanwhile, Tables 5–7 and Fig. 3 demonstrated the morphological characteristics of *F. solani*.

Morphological analysis of the conidial shapes of *F. oxysporum* were variable, elongated and straight to curve of macroconidia. While on microconidia, the shape was oval to kidney shape. The length × width of microconidia varied from 3.8–15.7 μm × 2.9–4.9 μm and that of macroconidia was around 14.8–38.5 μm × 2.4–5.7 μm. The number of septate was ranged from 1–5 septate. Chlamydospores were found formed as single and in pair. Chlamydospores are globose in shape and rough walled (Fig. 3). The colony growth rate of five *F. oxysporum* isolates on PDA were not significantly different, with isolate FO04 showing the highest growth rate mean (4.45 cm) on day 3 and isolate FO02 showing the least growth rate mean (3.27 cm) on day 3 (Table 2). Mycelia fully covering the plates within 5–7 days.

The colony of *F. solani* was fast growing covering the plate in 5–7 at 25°C PDA (Table 5). The colony growth rate of the 7 isolates on PDA were significantly different, with isolate FS02 showing the highest growth rate mean (4.20 cm) on day 3 and isolate FS06 showing the least growth rate mean (3.30 cm) on day 3. Mycelia were fully covering the plates within 5–7 days. Aerial mycelia are sparse, dense, floccose and abundant. The colony colour is white and white to light purple. Some isolates produce varies pigmentation (cream to yellow, salmon, yellow, yellow to orange and violet) and some of isolates did not produced any pigmentation (Table 6 and Fig. 3). Spodorochia covered by green masses of microconidia and some produced white spodorochia.

Morphological analysis of macroconidia shapes of *F. solani* were wide, straight to slightly curved, apical cell were rounded and basal cell have foot shape normally straight to cylindrical end. While microconidia shapes were oval, ellipsoidal or reniform. Chlamydospores were formed either singly or in pairs. The length x width of microconidia varied from 3.39–14.63 μm × 2.36–4.44 μm and that of macroconidia was around 7.22–50.46 μm × 2.43–6.14 μm. The number of septate was ranged from 1–5 septate (Table 7).

### Pathogenicity Test

**Figure 4:**
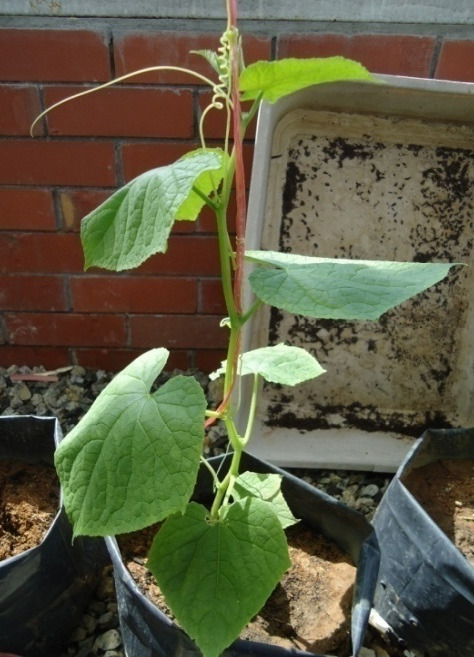

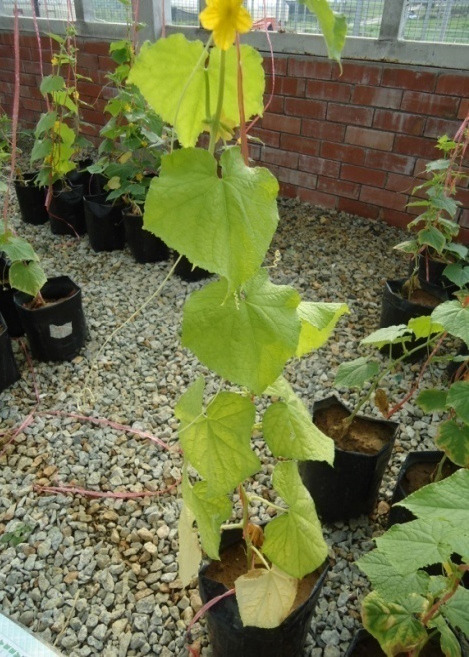

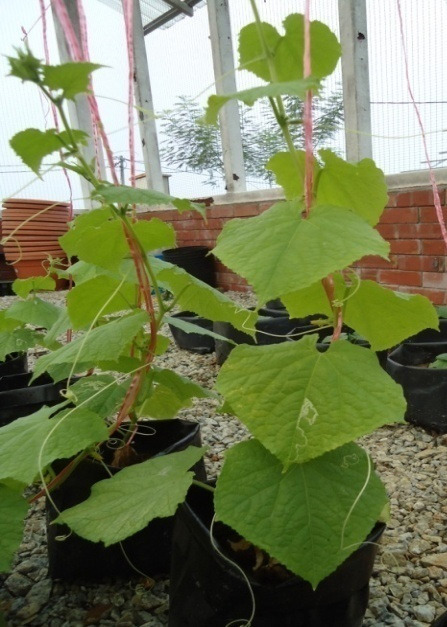
Various symptoms in *Fusarium* wilt disease in cucumber. (A) Infected cucumber plant showed early wilting symptom on leaves; (B) Cucumber plant showed severe yellowing and wilting of the leaves; (C) No symptom of *Fusarium* wilt disease on control treatment.

Pathogenicity study showed that both *Fusarium* species had self-evident effects on the growth of cucumber plants. Control plants showed no symptoms and remained healthy after four weeks of incubation period. Inoculated cucumber plants with *F. oxysporum* and *F. solani* showed similar disease symptoms which were leaf yellowing and wilt symptoms. The yellowing symptoms starts from old leaves, and then the leaves became chlorosis and wilted (Fig. 4). Our results showed that FS07 was the most aggressive isolate among *F. solani* with 44% of DSI and AUDPC value was 264 unit^2^. But, FS06 isolate was found to be the least aggressive among *F. solani* isolates with 8% DSI and AUDPC value was 53 unit^2^. For the negative control, the seedlings do not show any symptoms. Among *F. oxysporum*, FO03 isolate recorded the highest degree of aggressiveness as noted in the first week after inoculation (16% of DSI). Progressively, the DSI increased to 42% and AUDPC value was 259 units^2^ in the final week of assessment. Similarly, the negative control showed no disease symptom throughout assessment periods. Verification was done, whereby the infected cucumber plants produced *Fusarium* colonies after they were re-isolated to support Koch’s postulate. Details data of DSI and AUDPC of the tested fungal isolates were summarised in Table 8.

### Molecular Identification

**Figure 5:**
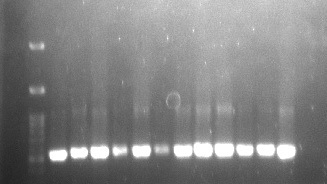
PCR products amplified with ITS1/ITS4 primers. Lane 1: 100bp DNA ladder; Lane 2: *F. oxysporum*; Lane 3–4: *F. solani*; Lane 5–6: *F. oxysporum*; Lane 7–8: *F. solani*; Lane 9: *F. oxysporum*; Lane 10–11: *F. solani*; Lane 12: *F. oxysporum*

In PCR study, DNA of *Fusarium* isolate was extracted using Qiagen DNeasy plant mini kit. The ITS region was amplified using pairs of universal primers ITS1 and ITS4. All the 12 isolates produced intense bands in between 300 bp–400 bp (Fig. 5). The ITS gene sequence of the 12 isolates were successfully obtained after being edited using Bioedit software (version 7.2). These sequences were deposited into Genbank database for validation. Based on BLAST analysis, the similarity index was ranging from 99%–100%. FO01–FO05 were confirmed to be *F. oxysporum* while, FS01–FS07 were *F. solani*. The details of the similarity index data were summarised in Table 9.

## DISCUSSION

Cucumber is a vegetable crop that can be grown all year-round in open field or glasshouse. However, this crop is highly vulnerable to fungal pathogens. Majdah (2015) reported that *Fusarium* wilt disease is the most destructive and commonly infecting cucumber crop. Without proper disease management will resulted huge yield loss. Results of the present study demonstrates that *Fusarium* wilt disease of cucumber in Peninsular Malaysia is caused by *F. oxysporum* and *F. solani*. Based on field survey data, the disease was highly prevalence (up to 60%) in the field, but no proper disease management was done by farmers and this is due to lack of scientific information. The cheapest and ideal detection approach that can be adopted by farmers in the field is based on field disease symptomatology.

In present study we confirmed that the disease symptoms were leaf yellowing and severe wilting on infected cucumber. Both pathogens exhibited similar disease symptoms in pathogenicity study, initially yellowing symptoms starts from old leaves, progressively the leaves will turn to chlorosis and finally the wilt symptom will be observed in all plant parts. Verification of the field detection and identification can be done by laboratory approaches.

Study by Champaco and Martyn (1993), revealed that *F. oxysporum* and *F. solani* showed symptoms of leaf yellowing and wilting of infected plants. Both disease symptoms were reported in this study. FO03 and FS07 isolates were the most aggressive *F. oxysporum* and *F. solani*, respectively. The widespread of these two isolates in cucumber farms could potentially cause a serious threat if there are no serious efforts done to control the disease in the field. According to Freeman and Rodriguez (1993), if cucumber plant had severe disease infection in early stage of growth and the plant will be wilted and finally died. This could suggest that cucumber plant had weak defend mechanism that cannot effectively defense them self against *Fusarium* from entering the vascular system. Furthermore, the root cells also failed to modify and limit the pathogen movement, thus it makes the plant susceptible to the pathogen attack (Majdah 2015).

Further study on cultural and morphological identification of the causative pathogens were conducted using various types of media. The most common media used for identification were PDA, Carnation Leaf Agar (CLA) and SNA (Leslie & Summerell 2006). In the present study, PDA and SNA were used. PDA was used for observation of morphological appearance and colony colouration, while SNA was for observation microscopic characterisation in identification of *Fusarium* spp. Initially, we had tried to culture the isolates on CLA media but was not successful as no microconidia, macroconidia and chlamydospores were produced. In the present study proved that SNA was found to be the best media for microscopic characterisation. SNA media was not decreasing the microscopic culture compared to other media. However, the media promotes sporulation and good conidiogeneous cell development similarly explained by Leslie and Summerell (2006). This result is supported by observation recorded by Iqbal *et al*. (2005), reported that SNA media is the best media to identify *F. mangiferae* because it can enhance production of microconidia and macroconidia. Moreover, report by Skovgaard *et al*. (2001) confirmed that SNA media is the best media to study of *F. oxysporum* complex isolated from soil and root necrosis of pea.

Morphological characteristics of *Fusarium* spp. plays very important roles in early stage for differentiation among various *Fusarium* species. This approach is considered less expensive but requires trained personal to do that. It is therefore the morphological identification of plant pathogenic fungi is the main approach and the most troublesome stride in recogniSable proof process particularly for *Fusarium* species (Rahjoo *et al*. 2008). Present study revealed that the morphological characteristics of *F. oxysporum* and *F. solani* isolated from infected cucumber were in line with the findings reported by Leslie and Summerell (2006) and Booth (1971). Based on the results, *F. oxysporum* showed variation in cultural and morphological characteristics and these findings agree with the work reported by Sonkar *et al*. (2014). Isolate FO01 produced white colony colour and pink pigmentation. *F. oxysporum* produced the aerial mycelium dense, floccose and abundant. The mycelium was white and later become purple. The orange spodorochia also produced. Morphological characteristics and microscopic characteristics agree with the previous report by Ibrahim *et al*. (2015). Abu Bakar *et al*. (2013), found that *F. oxysporum* pigmentation on PDA was white colour to purple, microconidia were having tapered apical cell and foot shaped of basal cell, microconidia were oval and kidney shaped with 0–1 septate. Based on the results of this study, *F. solani* isolates showed the aerial mycelium were sparse, dense, floccose and abundant. The colony colour produced were white to light purple. These characteristics were agreed to Sonkar *et al*. (2014).

Molecular identification research in Malaysia pertaining *Fusarium* spp. in cucumber is inadequate. Molecular study provides many important information for effective managing *Fusarium* wilt disease of cucumber. Polymerase chain reaction (PCR) assay have many advantages compared to conventional method in order to assess genetic variation in a wide range of pathogen. PCR technique is widely used for recognition of fungal pathogen in plant and soil. This was due to it cost effective, specificity, affectability and quickness (Zhang *et al*. 2012). PCR based techniques are likewise quick as there is no compelling reason to culture the *Fusarium* preceding their recognition (Arif *et al*. 2011). The key stride for the improvement of a PCR technique for pathogen detection is to outline aligonucleotide preliminaries. Internal transcribed spacer region of ribosomal DNA (rDNA) has been helpful to outline the PCR primers as a result of the high variability in ITS region among various species, empowering identification of fungal pathogens at the species level (Zhang *et al*. 2012). ITS gene arrangements are very variable among *Fusarium* species. ITS enhancement has been utilised to identify contagious pathogens for example *F. oxysporum* (Shahnazi *et al*. 2012). Ribosomal DNA (rDNA) in eukaryotes sowed a perfect focus for the recognition of various isolates/species of *Fusarium* (Dubey *et al*. 2014). Molecular segregation of *F. oxysporum* formae specials can even now be convoluted because of absence of learning encompassing the hereditary premise of virulence. This complexity within *F. oxysporum* has made it hard to find moderate qualities that are helpful as specific target successions to distinguish individual pathogenic formae specials (Scarlett *et al*. 2013). No report was found that using one step PCR method that able to detect and identify cucumber formae specials of *F. oxysporum* (Zhang *et al*. 2012). *Fusarium* wilts disease is highly difficult to manage without durably resistant cultivars. However, there are number of options are available and one of them is through integrated disease management approach. Although this technique may not be entirely effective but can help to lessen the severity of the disease in the field.

## CONCLUSION

*Fusarium* wilt of cucumber was highly prevalence in Peninsular Malaysia with field disease incidence in the range of 10%–60%. Pathogens identification was conducted through morphological, cultural and molecular approaches and had confirmed that the five isolates were *F. oxysporum* and seven isolates were *F. solani*. The pathogenicity test showed that the symptoms of *Fusarium* wilt disease was firstly appeared as yellowing of old leaves. Progressively, the infected plant will be wilted and finally died at four weeks after inoculation.

## Figures and Tables

**Table 1:** Type of field disease symptoms and disease incidence at the surveyed sites in Peninsular Malaysia.

State	Area	Symptoms	Disease incidence (%)
Selangor	Sepang	Wilt, leaf yellowing	15
Perak	Sungai Siput	Wilt	60
	Batu Gajah	Wilt	40
Pahang	Cameron Highland	Wilt	30
Johor	Kluang	Wilt, leaf yellowing	10

**Table 2:** Growth rate of *F. oxysporum* isolates at incubation period of 3, 5 and 7 days after incubation.

Isolate	Day 3	Day 5	Day 7
FO01	3.75b	5.78c	6.97b
FO02	3.27c	6.03c	7.45b
FO03	4.33a	7.25a	8.50a
FO04	4.45a	6.52b	7.50b
FO05	4.40a	7.03a	8.50a

*Note:* Means within rows followed by the same letter are not significantly different at *P* ≤ 0.01 by DMRT.

**Table 3:** Morphological characteristics of *Fusarium oxysporum* isolated from various locations in Peninsular Malaysia.

Isolate code	Location (city, state)	Conidia measurement (μm)				Number of septate

		Microconidia		Macroconidia		
	
		Length	Width	Length	Width	
FO01	Cameron Highland (Pahang)	7.67	3.31	20.99	3.93	1–3
FO02	Sungai Siput (Perak)	12.84	4.52	23.74	5.10	1–2
FO03	Batu Gajah (Perak)	15.66	4.91	38.54	5.77	2–3
FO04	Sepang (Selangor)	3.77	2.92	19.86	2.43	3–4
FO05	Sungai Siput (Perak)	6.63	2.54	16.36	3.12	1–2

*Note:* FO: *Fusarium oxysporum*. Number of septa, conidial length and width of 20 spores per each isolate.

**Table 4:** Differentiation among *Fusarium oxysporum* isolates based on colony morphology and pigmentation on PDA.

Isolates	Colony morphology	Colony colour	Pigmentation
FO01	Floccose and abundant	White	Pink
FO02	Dense and abundant	White	Violet and black concentric ring
FO03	Dense and abundant	White to light pink	Salmon
FO04	Floccose and abundant	White to salmon	Orange
FO05	Dense and abundant	White	Clear (No pigmentation)

*Note:* FO = *Fusarium oxysporum*

**Table 5:** The growth rate of *F. solani* isolates recorded at 3, 5 and 7 days after incubation period.

Isolate	Day 3	Day 5	Day 7
FS01	3.82b	6.58c	8.00b
FS02	4.20a	7.02ab	8.50a
FS03	3.00d	5.00e	6.55d
FS04	3.97b	7.27a	8.50a
FS05	3.45c	6.03d	7.50c
FS06	3.30c	5.98d	7.60c
FS07	3.92b	6.80bc	8.50a

*Note*: Means within rows followed by the same letter are not significantly different at *P* ≤ 0.01 by DMRT.

**Table 6:** Differentiation among *Fusarium solani* isolates based on colony morphology, colony colour and pigmentation on PDA.

Isolates	Colony morphology	Colony colour	Pigmentation
FS01	Floccose and abundant	White to light purple	Violet
FS02	Dense and abundant	White	Yellow
FS03	Floccose and abundant	White	Yellow to orange
FS04	Dense and abundant	White	Clear (No pigmentation)
FS05	Sparse	White	Clear (No pigmentation)
FS06	Floccose and abundant	White	Salmon
FS07	Dense and abundant	White	Cream to yellow

*Note:* FS: *Fusarium solani*

**Table 7:** Morphological characteristics of *Fusarium solani* isolated from various locations in Peninsular Malaysia.

Isolate code	Location (city, state)	Conidia measurement (μm)				Number of septate

		Microconidia		Macroconidia		
	
		Length	Width	Length	Width	
FS01	Cameron Highland (Pahang)	9.07	3.28	20.64	3.78	1–3
FS02	Sungai Siput (Perak)	6.94	2.36	14.45	2.94	1–3
FS03	Batu Gajah (Perak)	14.63	4.44	50.46	6.14	3–5
FS04	Sepang (Selangor)	3.77	2.92	19.86	2.43	1–3
FS05	Cameron Highland (Pahang)	3.39	2.83	7.22	2.69	1–3
FS06	Kluang (Johor)	9.62	3.52	18.70	4.32	1–3
FS07	Sungai Siput (Perak)	8.11	2.65	16.48	3.43	1–3

*Note:* FS: *Fusarium solani*. Number of septa, conidial length and width of 20 spores per each isolate.

**Table 8:** Aggressiveness of *Fusarium* spp. based on disease severity index (%) and AUDPC value after being inoculated on cucumber plants.

No	Isolates	Disease severity index (%)				AUDPC (units^2^)

		1WAI	2WAI	3WAI	4WAI	
1	FO01	0	4	14	34	121
2	FO02	6	10	34	36	219
3	FO03	16	18	32	42	259
4	FO04	0	4	8	24.4	120
5	FO05	12	14	20	30	177
6	FS01	2	6	8	18	76
7	FS02	14	14	28	48	244
8	FS03	12.5	15	32.5	42	250
9	FS04	10	10	18	32	161
10	FS05	2	4	8	16	67
11	FS06	0	6	8	8	53
12	FS07	6	16	38	44	264
13	Control	-	-	-	-	-

*Note*: WAI = week after inoculation

**Table 9:** Identified *Fusarium* species associated with *Fusarium* wilt of cucumber with their accession numbers and similarity index.

*Fusarium* species	Isolate code	Location	Accession number	Sequence with the best match	Similarity index (%)
*F. oxysporum*	FO01	Cameron Highland (Pahang)	KY307799	KY073258	100
*F. oxysporum*	FO02	Sungai Siput (Perak)	KY307805	KU097267	100
*F. oxysporum*	FO03	Batu Gajah (Perak)	KY307802	KT852968	99
*F. oxysporum*	FO04	Sepang (Selangor)	KY307809	JX519335	100
*F. oxysporum*	FO05	Sungai Siput (Perak)	KY307808	KT852968	99
*F. solani*	FS01	Cameron Highland (Pahang)	KY307803	KX064991	100
*F. solani*	FS02	Sungai Siput (Perak)	KY307800	KX064991	100
*F. solani*	FS03	Batu Gajah (Perak)	KY307801	KX650843	100
*F. solani*	FS04	Kluang (Johor)	KY307804	KU097246	99
*F. solani*	FS05	Cameron Highland (Pahang)	KX758537	KJ125723	100
*F. solani*	FS06	Kluang (Johor)	KY307807	KX064991	100
*F. solani*	FS07	Sungai Siput (Perak)	KY307806	KX171215	100

*Note*: Accession number equivalent obtained from GenBank database (http://www.ncbi.nlm.nih.gov/Blast).
